# The Emerging Role of Magnetic Resonance Imaging-Guided Focused Ultrasound in Functional Neurosurgery

**DOI:** 10.7759/cureus.9820

**Published:** 2020-08-17

**Authors:** Brian Fiani, India A Lissak, Marisol Soula, Kasra Sarhadi, Emad Salman Shaikh, Aqsa Baig, Mudassir Farooqui, Syed A Quadri

**Affiliations:** 1 Neurosurgery, Desert Regional Medical Center, Palm Springs, USA; 2 Physical Medicine and Rehabilitation, Spaulding Rehabilitation Hospital, Harvard Medical School, Boston, USA; 3 Medicine, New York University, New York, USA; 4 Neurology, University of Washington, Seattle, USA; 5 Neurology, Liaquat National Hospital and Medical College, Karachi, PAK; 6 Neurology, University of Iowa Hospitals and Clinics, Iowa City, USA; 7 Neurosurgery, Massachusetts General Hospital, Harvard Medical School, Boston, USA

**Keywords:** focused ultrasound (fus), high intensity focused ultrasound (hifu), magnetic resonance imaging-guided high-intensity focused ultrasound (mrghifu), thermal ablation, insightec neuro system, functional neurosurgery

## Abstract

Functional disorders of the central nervous system (CNS) are diverse in terms of their etiology and symptoms, however, they can be quite debilitating. Many functional neurological disorders can progress to a level where pharmaceuticals and other early lines of treatment can no longer optimally treat the condition, therefore requiring surgical intervention. A variety of stereotactic and functional neurosurgical approaches exist, including deep brain stimulation, implantation, stereotaxic lesions, and radiosurgery, among others. Most techniques are invasive or minimally invasive forms of surgical intervention and require immense precision to effectively modulate CNS circuitry. Focused ultrasound (FUS) is a relatively new, safe, non-invasive neurosurgical approach that has demonstrated efficacy in treating a range of functional neurological diseases. It can function reversibly, through mechanical stimulation causing circuitry changes, or irreversibly, through thermal ablation at low and high frequencies respectively. In preliminary studies, magnetic resonance imaging-guided high-intensity focused ultrasound (MRgHIFU) has been shown to have long-lasting treatment effects in several disease types. The technology has been approved by the FDA and internationally for a number of treatment-resistant neurological disorders and currently clinical trials are underway for several other neurological conditions. In this review, the authors discuss the potential applications and emerging role of MRgHIFU in functional neurosurgery in the coming years.

## Introduction and background

Functional neurosurgery is unique in its focus on altering specific central nervous system (CNS) circuitry. Altering circuitry enables clinicians to offer lasting treatments for diseases and disorders that are continually evolving [1-3]. This dynamic field of neurosurgery concentrates on new evolving technologies to improve and add precision and accuracy. The range of techniques utilized in functional neurosurgery are almost as diverse as the diseases they target, but most function through irreversible ablation or modulation. Current techniques include radiosurgery, deep brain stimulation, implantation and stereotaxic-guided ablation, among others. Many of the techniques used today are invasive/minimally invasive form of surgical intervention requiring immense precision to effectively modulate CNS circuitry.

High-intensity focused ultrasound (HIFU), when used with MRI for guidance termed as magnetic resonance imaging-guided high-intensity focused ultrasound (MRgHIFU) offers a highly precise, non-invasive alternative to current approaches by targeting deep brain structures without damaging the surrounding healthy tissue. The technology can be used to permanently ablate a target tissue without exposing the brain to the effects of ionizing radiation or to reversibly alter blood-brain barrier (BBB) permeability, enabling the delivery of therapeutic agents to targeted areas of the brain. The ultrasound waves cause thermal heating that gets absorbed locally, leading to tissue ablation. Alternatively, MRgHIFU can cause reversible modulation and therefore is uniquely positioned to enhance CNS pharmaceutical treatment by altering BBB permeability. Despite its promise, the technology is relatively young, and a number of clinical trials are still ongoing across disease types (Table 1). This review aims to increase awareness of HIFU by describing the mechanisms of action as well as current and future applications in functional neurosurgery. We explore the use of MRgHIFU to treat a variety of disorders including neuropathic pain, essential tremor, Parkinson’s disease, obsessive compulsive disorder, depression, trigeminal neuralgia, and epilepsy. Furthermore, we review applications for MRgHIFU in temporarily altering BBB permeability for improved pharmaceutical intervention.

**Table 1:** Clinical trials studying magnetic resonance imaging-guided high-intensity focused ultrasound (MRgHIFU)

Neurological Disease	Trial Status	Country	Study
Chronic Neuropathic Pain	Ongoing Clinical Trial	United States	Targeting central lateral thalamus for treating neuropathic pain
	Ongoing Clinical Trial	United States	For treatment refractory, chronic trigeminal neuropathic pain
	Ongoing Clinical Trial	Israel	For ablation of painful neuromas causing amputee stump pain
Essential Tremor	FDA approved	United States	For medication-refractory essential tremor patients
	Ongoing Clinical Trial	UK and Spain	Treatment of Medication Refractory Essential Tremor
Parkinson’s Disease	Ongoing Clinical Trial	United States	For Medically-Refractory Dyskinesia Symptoms or Motor Fluctuations of Advanced Parkinson’s Disease
	Ongoing Clinical Trial	China and Japan	For the Treatment of Parkinson’s Disease
	Ongoing Clinical Trial	Canada	Unilateral Pallidotomy for the Treatment of Parkinson’s Disease
Obsessive Compulsive Disorder	Ongoing Clinical Trial	Canada	Bilateral Capsulotomy for the Treatment of Refractory Obsessive-compulsive Disorder
Major Depressive Disorder	International Approval	Korea	Treatment of Major Depressive Disorder via Magnetic Resonance-Guided Focused
	Ongoing Clinical Trial	Canada	Ultrasound Surgery - MgFUS for Treatment of Medically Refractory Depression
Trigeminal Neuralgia	Ongoing Clinical Trial	United States	Thalatomy for Treatment of Chronic Trigeminal Neuropathic Pain
Epilepsy	Ongoing Clinical Trial	United States	For medication-refractory epilepsy with subcortical focal lesions
	Ongoing Clinical Trial	United States	For drug resistant temporal lobe epilepsy
	Ongoing Clinical Trial	United States	To prevent the secondary generalization from focal onset epilepsy
	Ongoing Clinical Trial	United States	For medication-refractory epilepsy in patients with subcortical lesions

## Review

Historical perspective

High intensity focused ultrasound (HIFU) was born when John Lynn and colleagues constructed a focus ultrasound generator that destroyed brain tissue without damaging skin or surrounding tissue [4]. In 1946, William and Francis Fry designed focus piezoelectric transducers that could control the ultrasound (US) beam to more precisely target lesions [5]. HIFU takes advantage of the increased sensitivity to hyperthermia in tumor microenvironments relative to normal tissue that can therefore be selectively lesioned [6-10].

**Figure 1:**
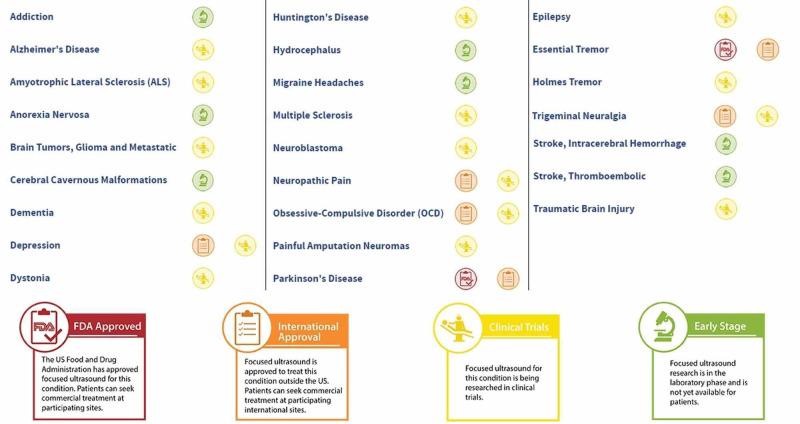
Current neurological applications of high-intensity focused ultrasound in various stages of research and approvals. (Credit: Image obtained from the Focus Ultrasound Foundation)

Diagnostic ultrasound imaging was initially used alongside HIFU, however, in the early 2000s, utilization of Magnetic Resonance Imagining (MRI) increased guidance precision and provided a mechanism for measuring temperature in real-time. With better intraoperative visualization techniques and MRI thermometry, HIFU has become much safer [11-15]. Given the demonstrated precision of HIFU, its usage has been explored as an avenue for treatment in an array of disease types. In the realm of functional disorders, MRI combined with guided focused ultrasound (MRgHIFU) has been used to treat Parkinson’s disease, movement disorders, neuropathic pain, stroke, and, most recently, epilepsy and obsessive-compulsive disorder [12,16-19]. The stages of approval for various CNS disorders are depicted in Figure 1.

Principles and mechanism of action

**Figure 2:**
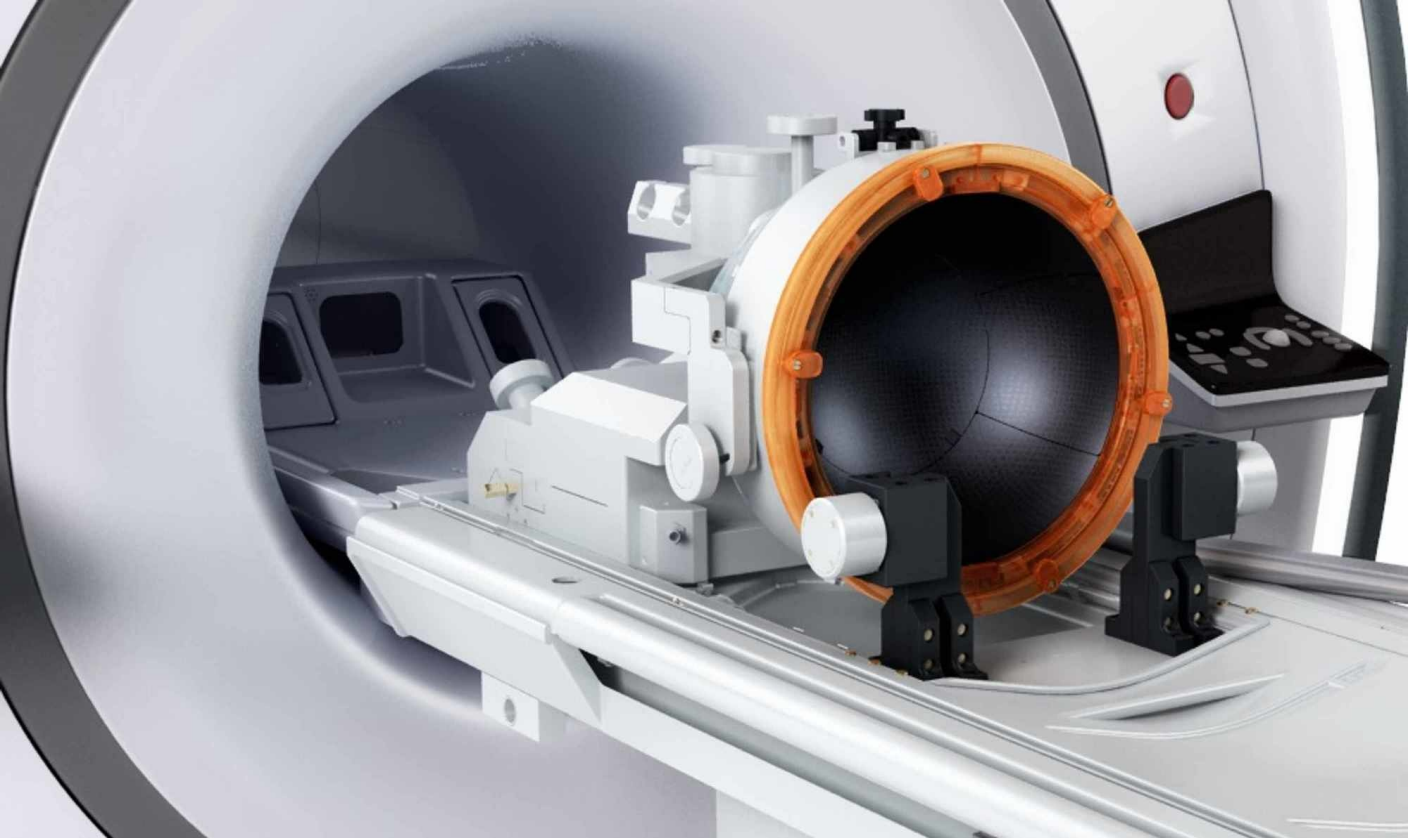
The Phase array, piezoelectric helmet shaped high-intensity focused ultrasound transducer with 1024 elements at a frequency of 650 KHz. (Credit: Image obtained from INSIGHTEC Ltd.)

MRgHIFU functions by concentrating high-energy US waves onto a target and inducing localized thermal ablation. Piezoelectric transducers focus the US energy onto the tissue of interest (Figure 2). The “focal zone,” is the region where the US intensity (energy/unit area), usually in the range of 100-10,000 W/cm2, is large enough to produce a lesion. Temperature and duration of exposure determine the resulting lesion size. The US energy creates a series of reactions on biological tissues that allow for specific ablation of target tissues, like tumors, while preserving neighboring structures [11,20].

Thermal Mechanisms and Non-thermal (Mechanical) Mechanisms of Action

The two main mechanisms involved in MRgHIFU include thermal and non-thermal (mechanical) reactions on biological tissues. The thermal effect is mitigated by the conversion of US energy into frictional heat, resulting in temperature increases to around 55 degrees Celsius. These temperature changes damage the cytoplasm and cell membrane, ultimately leading to cellular death and tissue destruction. Histologically, the destroyed tissue comprises an “island and moat” formation where the island represents the coagulation necrosis and the moat represents a rim of damaged glycogen depleted cells destined for death [21]. Within seven days, phagocytes and leukocytes begin to surround the US damaged cells and eventually the granulation tissue is deposited around the lesion, leaving behind scar tissue [21].

The non-thermal mechanism involved in MRgHIFU ablation, inertial cavitation (IC), is created by the delivery of US in pulses. The rapid changes in pressure caused by these pulses leads to the formation of unstable bubbles in the liquid medium found in tissues [22]. Turbulence generated by bubble formation and collapse drives mechanical tissue destruction, particularly in the extracellular space.

Attenuation of Energy

When US energy travels through a medium, its amplitude attenuates as mechanical energy is converted into thermal energy. The attenuation coefficient of a particular medium is important to consider as tissues with large attenuation coefficients pose a challenge for MRgHIFU. For example, bone has a high attenuation coefficient (6.9 dB/cm MHz) compared to muscle tissue (1.09 dB/cm MHz) and, as a result, it absorbs and reflects a significant portion of US energy (frequency range of 3-12 MHz) [23]. This leads to MRgHIFU beam defocusing and overheating of the skull. A skull cooling system and a piezoelectric transducer that prevents scatter helps to avoid excessive heat transfer to the skull and target specific brain areas with limited injury to the surrounding area. It is important to monitor the attenuation coefficient during MRgHIFU therapy because it increases as the temperature rises [24].

Challenges with transcranial MRgHIFU

The greatest challenge posed by MRgHIFU is related to energy attenuation in dense tissues. Regions with large attenuation coefficients, like the skull, can cause undesirable heating, reflective tissue damage of the skin and dissipation of US energy [23]. While this was once considered a serious concern, the use of external cooling systems, high energy sources, and CT-driven transducer arrangements has nearly eliminated this problem.

Appropriately focusing US beams to avoid unwanted biological reactions is another technical challenge posed by MRgHIFU. This technical challenge has been partially mitigated by using multiple sources of US waves with various geometric lenses for focusing. Another alternative is to alter the temporality of US waves from different transducers thereby enabling beam-steering and precise focusing on the target region. At present, most transducers create ellipsoid (cigar) shaped tissue lesions [21]. Creating transducers with additional shapes would allow us to alter focal regions to target clinically relevant volumes.

Current applications in functional neurosurgery

Chronic Neuropathic Pain

Chronic neuropathic pain is a medical problem that plagues millions of Americans. The etiology and pathophysiology are often complex and difficult to pinpoint, posing a unique challenge for medical practitioners. Though a variety of medical therapies for chronic neuropathic pain do exist, including NSAIDs, sodium-channel blockers, anti-epileptic drugs, and procedural nerve blocks, these therapies are not universally effective. Some studies report that only half of neuropathic pain patients experience a 30-50% relief with the current therapeutic options [25].

Neurosurgical interventions have been used in patients with chronic neuropathic pain refractory to other therapies. Common procedures target the centrolateral thalamus via radiofrequency (RF) ablation, radiosurgery, or deep brain stimulation (DBS). MRgHIFU may offer an equally effective, non-invasive alternative. In 2009, Martin et al. conducted centrolateral thalamotomies via MRgHIFU in nine patients with a variety of pain etiologies [26]. All subjects reported immediate pain relief during sonication, a 68% mean pain reduction 48 hours after treatment, and a 57% mean pain relief one year post-treatment. Jeanmonod et al. (2012) reported neuropathic pain relief in 11 patients treated with MRgHIFU by 49% at three months post-treatment and 57% at one year [27].

These studies suggest that MRgHIFU is an effective treatment for reducing chronic neuropathic pain. It is important to note, however that a thalamic hemorrhage resulting in neurological deficits did occur in one subject. Additional protocol precautions involving measures to detect cavitation and limiting the thermogenesis of sonication to less than 60°C were subsequently implemented [16]. While these studies were small, they suggest that MRgHIFU does offer an effective non-invasive method for treating refractory chronic neuropathic pain. The use of MRgHIFU ablation for chronic pain has already been approved in several other areas of the world such as Israel, Europe, Canada, Japan, China, Russia, Korea, Brazil, India, and Australia, and clinical trials in the United States are ongoing [25].

Essential Tremor (ET)

Essential tremor (ET) is the most prevalent movement disorder, affecting nearly 2.2% (>7.5 million) of the US population [28]. Clinical symptoms can be quite debilitating and include upper extremity or axial tremor that persist at rest and worsen with movement. First-line medical therapy for ET usually involves beta blockers (like propanolol) or barbiturates (like primadone), but approximately 30% of individuals with ET do not respond to medication, and thus require unique treatments [29]. In fact, deep-brain stimulation (DBS) was first used to treat ET through targeting of the ventral intermediate nucleus (VIM) of the thalamus [14]. The success of DBS in reducing ET symptoms paved the way for the first clinical application of MRgHIFU in the treatment of ET.

Several pilot studies utilizing MRgHIFU to target the VIM reported contralateral tremor improvements ranging from 75-81% with minimal adverse effects [12,14,30,31]. In 2013 Elias et al. reported treating 15 patients with drug resistant ET using transcranial MRgHIFU for ablation of the unilateral VIM of the thalamus between February 2011 to December 2011. At one-year follow-up, significant improvement was noticed in tremors (p=0.001), total tremor scores (p=0.001), disability scores (p=0.001), and quality of life scores (p=0.001) in comparison with the preoperative scores. Adverse effects noted were transient sensory, motor, speech, and cerebellar abnormalities, with four patients developing permanent paresthesias [12]. The same year Lipsman et al. also reported treating four patients with ablation of the thalamic focus of the ET, resulting in a mean reduction in tremor scores of 81.3% at three months compared with baseline [30].

In 2016, Gallay et al. also reported satisfactory results for transcranial MRgHIFU cerebellothalamic tractotomy in 21 patients with ET [31]. The same year Elias et al. reported their randomized controlled trial using MRgHIFU for thalamotomy versus a sham procedure [13]. Hand-tremor scores improved in transcranial MRgHIFU thalamotomy patients (from 18.1 points at baseline to 9.6 at three months), with a between-group difference in the mean change of 8.3 points (95% CI 5.9-10.7, p<0.001).

Kim et al. presented a comparative evaluation comprising of 59 patients who underwent MRgHIFU (n=23) versus RF thalamotomy (n=17) or DBS (n=19) for ET treatment [32]. No statistical differences in treatment success were observed between the three treatment groups over time. However, complication rates differed between treatment modalities (p<0.01) and were lowest in the MRgHIFU group.

A 76-patient, multicenter randomized trial reported results at a two-year follow-up [33]. The results revealed a significant benefit compared to a control group with a 47% improvement in hand tremor scores at three months and a 40% improvement at one year following treatment. Mean hand tremor score at baseline (19.8 ± 4.9; 76 patients) improved by 56% at two years (8.8 ± 5.0; 67 patients). Similarly, the disability score at baseline (16.4 ± 4.5; 76 patients) improved by 64% at two years (6.5 ± 5.0; 67 patients). Although adverse effects including paresthesia and balance instability were reported, the side effects diminished over time and did not appear to impact the subjects’ perceived post-sonication quality of life improvement. No further new delayed complications were reported at the two-year mark [33]. The results of these studies led to the first FDA approval of MRgHIFU in 2016 for the treatment of ET [13,33].

Parkinson's Disease (PD)

Parkinson's Disease (PD) is a degenerative disease of the dopaminergic tracts of the basal ganglia, affecting 0.15% of the population and 1.5% of individuals over the age of 70 [16]. Notable symptoms include resting tremor, rigid extremities, akinesia, postural instability, and shuffling gait. Levodopa is the primary medical therapy for PD, however, the latent “on-off” effects of long-term levodopa use cause the therapeutic window to diminish over time [16]. This decline in levodopa efficacy over time led clinicians to pursue longer-lasting treatments for PD, like DBS of the subthalamic nucleus. PD researchers turned to MRgHIFU as a durable non-invasive alternative to DBS for treating PD.

In 2014, a studt utilizing MRgHIFU to target the tract between the globus pallidus and thalamus demonstrated a 60.9% improvement according to the Unified Parkinson Disease Rating Scale (UPDRS) and a 56.7% improvement in global symptom relief (GSR) at three months post-ablation in nine patients receiving five to six rounds of sonication compared to only a 7.6% improvement in UPDRS and 22.5% improvement in GSR in the group receiving only one round of sonication [14,25,34]. MRgHIFU was subsequently used successfully with VIM thalamotomy for patients with PD with minor side effects including instability in walking, hand ataxia and taste disturbances [14,35,36]. A randomized control trial by Bond et al. reported significant betterment in hand tremors and UPDRS scores in PD patients with tremors treated with MRgHIFU [37]. While the only case of pallidotomy was reported by Na et al. in a woman with levodopa associated motor dysfunction [38].

MRgHIFU provides a unique non-invasive alternative to surgical intervention. At present, its use to treat medication-refractory PD is approved in Israel, Europe, Korea, Russia and tremor-predominant PD in US [39]. Furthermore, a multi-center clinical trial is currently underway to evaluate the safety and efficacy of unilateral pallidotomy to improve medically-refractory dyskinesias or motor fluctuations in advanced PD [39,40]. Another ongoing study in Spain is investigating unilateral subthalamotomy for motor symptoms of PD such as slowness and stiffness [41]. Alternative usage for MRgHIFU in PD treatment is also being explored as a mechanism for increasing BBB permeability to enhance medication localization in the CNS [25,42].

Obsessive-Compulsive Disorder (OCD) and Major Depressive Disorder (MDD)

Obsessive-compulsive disorder (OCD) and major depressive disorder (MDD) have high rates of recurrence following medical therapy. The Sequenced Treatment Alternatives to Relieve Depression (STAR*D) study estimated that one-third of the 3671 participants failed several attempts at medical therapy [43]. Like other functional disorders, neurosurgical interventions like DBS have been shown to effectively treat medically refractory cases.

Ablation targets for surgical treatment of OCD and MDD include areas largely contained within the limbic system, like the anterior limb of the internal capsule (ALIC), anterior cingulate cortex, subgenual cingulate cortex, and ventral striatum [25]. A 2015 study by Jung et al. was the first to use MRgHIFU to treat OCD [11]. A bilateral capsulotomy of the ALIC was carried out with MRgHIFU in four patients with medically-refractory OCD [11]. While the bilateral ALIC capsulotomy was conducted to improve OCD symptoms, the treatment had a more pronounced effect on relieving MDD in the three comorbid patients [11]. According to the Yale-Brown Obsessive-Compulsive Scale, OCD symptoms improved by an average of 33% at six months following treatment. In contrast, depression symptoms, measured by the Hamilton Depression Rating Scale, improved an average of 61.1% at six months. Treatment-related adverse effects were minimal. Rates of symptom improvement persisted for both OCD and MDD at two years following MRgHIFU [25].

A clinical trial utilizing MRgHIFU to target bilateral ALIC for treatment-refractory OCD and MDD is underway [44]. Further clinical exploration is warranted to assess whether MRgHIFU could alternatively be used to transiently disrupt the blood-brain barrier in order to enhance CNS uptake of antidepressant medications [45].

Trigeminal Neuralgia

Trigeminal neuralgia is a rare, often debilitating, disease with an incidence of four to 13 people per 100,000 [46]. It is characterized by electrical-like facial pain occurring unilaterally in branches of the trigeminal nerve that results from classically nonpainful stimuli. The administration of one or more antiepileptic drugs like carbamazepine, oxcarbazepine, or lamotrigine is usually the first-line of treatment. Few surgical options for refractory trigeminal pain have been explored, but the procedures that have been include highly invasive ablative, decompressive, or resection approaches. Gamma knife radiosurgery has shown promise as a non-invasive tool for treating refractory trigeminal neuralgia. A systematic review reported full symptom relief in 75% of patients within three months of treatment and 50% with sustained relief at three years [46].

Clinical trials evaluating the safety and efficacy of MRgHIFU as a treatment for trigeminal neuralgia are yet to be carried out. There is some concern about using MRgHIFU due to the depth of the trigeminal nerve and therefore the potential damage to surrounding structures. However, a cadaveric feasibility study targeting the proximal trigeminal nerve reported that MRgHIFU was safe and effective [47]. The study implemented “no-pass” regions, areas specifically excluded from the path of sonication, which helped to minimize heating of the skull base and surrounding structures while still successfully ablating the trigeminal nerve [47]. Additional safety and efficacy studies are required to determine whether implementing MRgHIFU may be useful as a treatment of medication-refractory trigeminal neuralgia.

Neuromodulation and Epilepsy

There are seemingly endless possibilities for how ultrasound technology can be utilized to modify neuronal activity on a cellular level in the CNS or PNS. Ultrasound technology could provide a non-invasive technique for altering brain function, reversibly, through mechanical stimulation forces altering voltage-gated ion channels, or irreversibly, through ablation. Early studies in animals demonstrate that brief sonication pulses delivered by low-intensity focused ultrasound (LIFU) can alter the activity in specific cortical regions without the irreversible thermal effects of HIFU. Examples include inducing activity in hippocampal regions, stimulating motor responses in rodents, and altering frontal eye field responses in primates [14]. In human studies of LIFU, successful stimulation of the primary somatosensory cortex resulted in alterations in the perception of tactile sensation [48,49].

MRgHIFU may also have similar neuromodulatory applications. One disease subtype in particular that could benefit from MRgHIFU as a neuromodulatory treatment is refractory epilepsy. Nearly 20-40% of individuals with epilepsy do not respond to antiepileptic drugs and therefore require surgical intervention [25]. Before non-invasive neurosurgical technologies like MRgHIFU were used, the surgical treatment for epilepsy included lobectomy/lesionectomy, stereotactic radiosurgery and laser interstitial thermotherapy [25]. Though less invasive, the two latter approaches, stereotactic radiosurgery and laser interstitial thermotherapy, still carried significant risks with ionizing radiation (for radiosurgery) and scalp incision/trephination (for laser interstitial thermotherapy). Studies using rodent models to investigate use of MRgHIFU for treating epilepsy found that low-powered pulses delivered to the thalamus reduced pentylenetetrazole-induced epileptic activity by 30% [16]. Human studies are currently ongoing to study the feasibility of utilizing MRgHIFU for treating epilepsy. Ablative approaches, caused by subcortical lesions like hypothalamic hamartomas and tuberous sclerosis, as well as non-ablative neuromodulation, for treating temporal lobe epilepsy, are being investigated [25].

Blood-Brain Barrier Modification

The blood-brain barrier (BBB), a semipermeable membrane made up of endothelial tight junctions, lines the cerebral vasculature to prevent substances from readily passing into cells from the intravascular space. While it does serve an extremely important function by preventing toxins and other damaging substances from entering the brain, it also poses a challenge when designing medications as it can be difficult to create pharmaceuticals that cross the BBB to induce their desired effect.

The enhancement of molecule delivery through the BBB via temporary increases in permeability is another promising application for MRgHIFU. Early studies have shown potential by introducing an oscillating microtubule contrast agent that, when pulsed via sonication, created a shearing stress that induced a reversible, spatially specific, increase in BBB permeability [25]. There have been several studies of MRgHIFU use in animals resulting in similarly increased BBB permeability for the delivery of chemotherapeutic agents, antibodies, and other neurotrophic factors into the CNS [39]. For example, a 13- to 14-fold increase in CNS chemotherapeutic drug concentrations after utilizing MRgHIFU to induce BBB-opening was discovered in rabbit models [25]. In humans, a Phase I clinical trial using MRgHIFU to increase BBB permeability in patients with Alzheimer’s Disease was recently completed [50]. While the results of the trail have yet to be reported, if successful, the use of this technology to introduce anti-amyloid antibodies and enhance the delivery of other disease-modifying drugs could signify an incredible advancement in the treatment of neurodegenerative diseases. Additional studies are required to establish the viability of using MRgHIFU to modulate BBB permeability for treating diverse neurological and oncological diseases.

## Conclusions

MRgHIFU offers a non-invasive alternative to many functional neurosurgical techniques that are presently being used. This technique has the potential to effectively treat a range of debilitating diseases and disorders without carrying many of the same risks or side effects as other more invasive approaches. MRgHIFU treatment has been approved for usage in the US and internationally for the treatment of several diseases, however, additional human trials are required to establish safety and efficacy in others.
